# Video Monitoring a Simulation-Based Quality Improvement Program in Bihar, India

**DOI:** 10.1016/j.ecns.2017.11.007

**Published:** 2018-04

**Authors:** Jessica Dyer, Hilary Spindler, Amelia Christmas, Malay Bharat Shah, Melissa Morgan, Susanna R. Cohen, Jason Sterne, Tanmay Mahapatra, Dilys Walker

**Affiliations:** a Program Director, PRONTO International, Seattle, WA 98112, USA; b Project Director, Institute for Global Health Sciences, University of California San Francisco, San Francisco, CA 94158, USA; c Senior Simulation Specialist, Pronto International, State RMNCH+A Unit, Patna, Bihar, India; d CARE India Solutions for Sustainable Development, Bihar Technical Support Unit, Bihar, India; e Institute for Global Health Sciences, University of California San Francisco, San Francisco, CA 94158, USA; f Assistant Professor of Neonatology, Department of Pediatrics, University of California San Francisco, San Francisco, CA 94158, USA; g Maternal, Adolescent, Reproductive, & Child Health Centre, London School of Hygiene & Tropical Medicine, Keppel Street, London, UK; h Associate Professor, College of Nursing, University of Utah, Salt Lake City, UT 84112, USA; i Chief Operations Officer, PRONTO International, Seattle, WA 98112, USA; j Team Lead, CARE India Solutions for Sustainable Development, Bihar Technical Support Unit, Bihar, India; k Professor, Department of Obstetrics and Gynecology and Reproductive Services, University of California San Francisco, San Francisco, CA 94110, USA

**Keywords:** maternal and child health, simulation training, program monitoring, quality improvement, video monitoring

## Abstract

**Background:**

Simulation-based training has become an accepted clinical training andragogy in high-resource settings with its use increasing in low-resource settings. Video recordings of simulated scenarios are commonly used by facilitators. Beyond using the videos during debrief sessions, researchers can also analyze the simulation videos to quantify technical and nontechnical skills during simulated scenarios over time. Little is known about the feasibility and use of large-scale systems to video record and analyze simulation and debriefing data for monitoring and evaluation in low-resource settings.

**Methods:**

This manuscript describes the process of designing and implementing a large-scale video monitoring system. Mentees and Mentors were consented and all simulations and debriefs conducted at 320 Primary Health Centers (PHCs) were video recorded. The system design, number of video recordings, and inter-rater reliability of the coded videos were assessed.

**Results:**

The final dataset included a total of 11,278 videos. Overall, a total of 2,124 simulation videos were coded and 183 (12%) were blindly double-coded. For the double-coded sample, the average inter-rater reliability (IRR) scores were 80% for nontechnical skills, and 94% for clinical technical skills. Among 4,450 long debrief videos received, 216 were selected for coding and all were double-coded. Data quality of simulation videos was found to be very good in terms of recorded instances of “unable to see” and “unable to hear” in Phases 1 and 2.

**Conclusion:**

This study demonstrates that video monitoring systems can be effectively implemented at scale in resource limited settings. Further, video monitoring systems can play several vital roles within program implementation, including monitoring and evaluation, provision of actionable feedback to program implementers, and assurance of program fidelity.

## Highlights

• We collected 11,657 simulation and debrief videos at 320 primary health centers in Bihar, India.
• Video monitoring can support monitoring/evaluation and actionable feedback and assure fidelity.
• Video monitoring systems can be effectively implemented at scale in resource-limited settings.

**Key Points**

• We collected 11,278 simulation and debrief videos at 320 primary health centers in Bihar, India.
• Video monitoring can support monitoring/evaluation and actionable feedback and assure fidelity.
• Video monitoring systems can be effectively implemented at scale in resource-limited settings.

Simulation-based training has become an accepted clinical training andragogy in high-resource settings, providing trainees the opportunity to practice skills necessary to effectively manage rare but serious emergencies. Simulation effectively improves technical and nontechnical skills of health care providers in a variety of disciplines (Bragard et al., 2016, Davis et al., 1999, Kim and Shin, 2016, Walker et al., 2016). As innovations continue, simulation now reaches areas with limited resources and equal if not greater needs for improved quality of care. In recent years, the use of high-fidelity simulation has increased in low-resource settings with promising results (Walker et al., 2014, Walker et al., 2016).

Video recordings of simulated scenarios are commonly used by facilitators during debriefing sessions to provide factual documentation of the simulation, guide conversation, and stimulate reflection and self-guided learning (Sawyer, Eppich, Brett-Fleegler, Grant, & Cheng, 2016). Video playback reduces recall bias by providing evidence of actions during the simulated scenario, thus allowing participants to see how they performed rather than how they thought they performed (Levett-Jones et al., 2014). Beyond using the videos during debrief sessions, for large-scale projects, researchers can also analyze the simulation videos to quantify technical and nontechnical skills during simulated scenarios over time. Systems to video record simulations for use in both video-guided debriefing and implementation research are useful for monitoring and evaluating simulation-based programs. Furthermore, video recordings of debriefing sessions also assist programs in monitoring debriefing quality and facilitator performance progressively.

Little is known, however, about the feasibility and use of large-scale systems to video record and analyze simulation and debriefing data for monitoring and evaluation in low-resource settings. Ongoing collection of video data to monitor and evaluate maternal and child health simulation-based training programs is needed to ensure program fidelity, show efficacy, and target limited resources. Low-resource settings present unique challenges when using video-based monitoring and evaluation systems at scale because of poor Internet connectivity, remote locations, and infrastructure issues. Direct observation of deliveries, considered the gold standard for showing changes in clinical practice from training, is expensive and often does not capture data on clinical performance during emergency scenarios because of their relative rarity (Tripathi et al., 2015).

To provide facilitators the ability to conduct video-guided debriefs and measure changes in technical and nontechnical skills of mentees, we developed a large-scale video monitoring system that evaluates both simulations and debriefs in Bihar, India. The aim of the overall study was to examine changes in technical and nontechnical skills in simulated clinical scenarios in a large-scale quality improvement project. This article describes the feasibility and design of the video monitoring system and the coding process.

## Methods

### Setting

The project was implemented in 320 primary health centers (PHCs) in Bihar, India, from January 2015 to January 2017. Bihar's approximate population of 115 million makes it the third largest state in India (Primary Census Abstract Data Highlights - 2011 India & States, 2011), with approximately 524 PHCs statewide (District Level Household and Facility Survey (DLHS-4), 2012-2013, 2014). In 2012 to 2013, the state reported a maternal mortality ratio of 208 (163-253) maternal deaths per 100,000 live births (MMR Bulletin Sample Registration System, 2013) and a neonatal mortality rate of 34 neonatal deaths per 1,000 live births (District Level Household and Facility Survey (DHLS-4), 2012-2013, 2014), both significantly higher than the averages in India. PHCs are state-owned rural health care facilities typically staffed with a single medical officer and several auxiliary nurse midwives and general nurse midwives.

### Intervention

In 2015, CARE India (CARE), in collaboration with the Government of Bihar, launched a program called Apatkalin Matritva evam Navjat Tatparta translating to emergency obstetrical and neonatal readiness. One goal of this comprehensive system strengthening and quality improvement initiative was to improve maternal and neonatal health outcomes in public health facilities through interventions aimed at improving supply procurement, provider skills and behavior, and quality of clinical care through adult learning techniques (Das et al., 2016).

In 2014, the University of California, San Francisco (UCSF), PRONTO International (PRONTO) (www.prontointernational.org), and the University of Utah partnered with CARE India and the Government of Bihar to integrate PRONTO's highly realistic simulation and team training activities into the Apatkalin Matritva evam Navjat Tatparta program through mobile nurse midwife mentoring at 320 PHCs by a cohort of 120 nurse mentors (NMs), brought into Bihar mostly from outside the state. The aim was to improve the skills of auxiliary nurse midwives/general nurse midwives (mentees) working at the PHC level. PRONTO, in partnership with the University of Utah, developed a Bihar-specific simulation and team training curriculum to complement CARE India's curriculum. The NMs were trained as mentors by CARE and PRONTO. PRONTO trained the NMs to run simulations, facilitate video-guided debriefing sessions, and conduct postevent debriefing after live births. The simulation and team training activities provided mentees with opportunities to practice technical competencies for the management of a variety of neonatal and obstetric emergency cases as well as nontechnical competencies for better teamwork and communication.

**Figure 1:**
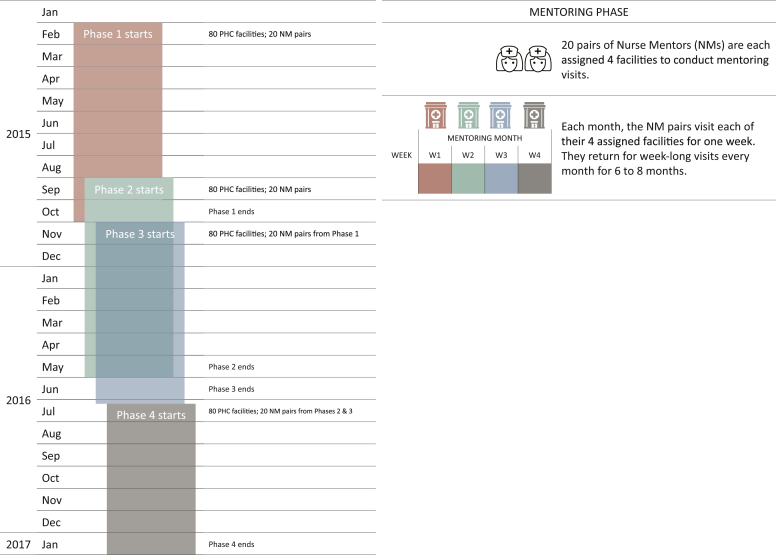
Mobile nurse mentoring program implementation timeline.

Pairs of NMs were assigned four PHCs to conduct on-site mentoring visits. Each month, NM pairs visited their four assigned facilities for one week. They returned for weeklong visits every month for eight months. The program was implemented in four phases at 80 facilities per phase (Figure 1). During each weekly visit, NMs were instructed to facilitate simulations for at least three specified clinical scenarios: normal spontaneous vaginal deliveries (NSVDs) as well as common preventable causes of morbidity and mortality including postpartum hemorrhage (PPH) and neonatal resuscitation (NR) for birth asphyxia. To reproduce the environment in which the mentees work as closely as possible, NMs were instructed to conduct simulations in the labor room as often as possible. Most frequently, these in situ simulations were conducted with a mentee playing the part of the mother, wearing PartoPants™ (Laerdal Global Health, Stavanger, Norway), a hybrid birth simulator, and using a NeoNatalie^®^ (Laerdal Global Health, Stavanger, Norway) infant manikin to simulate NR.

In addition, at two points during each phase, midpoint (after week 3 of mentoring) and endpoint, mentees participated in three simulations (NSVD, PPH, and NR) where performance was assessed to objectively measure changes over time.

### Data Collection

**Figure 2:**
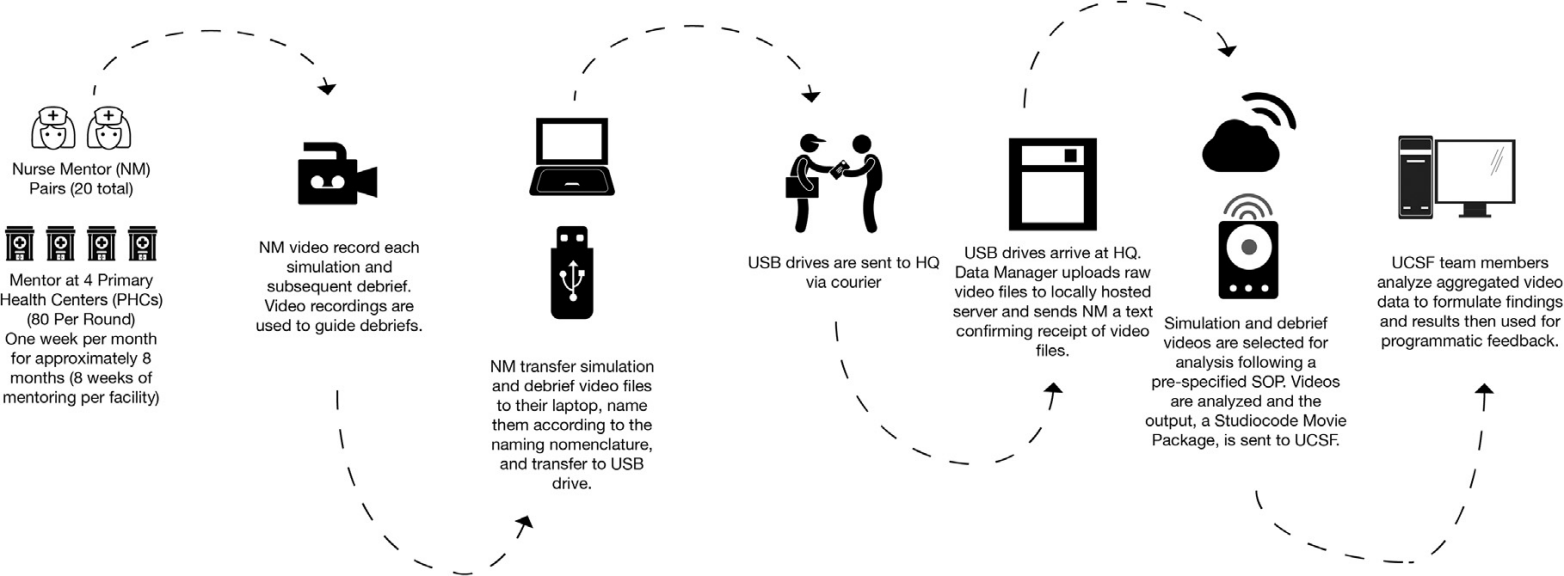
Video data collection and management process in Bihar, India.

NMs video recorded every simulation and debrief completed at the PHCs on video cameras provided by the program. Video footage was renamed using a prespecified nomenclature, uploaded to a computer by the NMs as a mp4 video, transferred to encrypted universal serial bus drives, and sent to headquarters in the state capital, Patna, via courier. Video footage was transferred to a secure server by the data manager. The data manager followed a standard operating procedure of video selection to distribute video files to two Hindi-speaking video analysts (VAs) with four-year undergraduate degrees in nursing for video coding. Figure 2 captures the data collection and management process.

### Video Monitoring System Development

**Figure 3:**
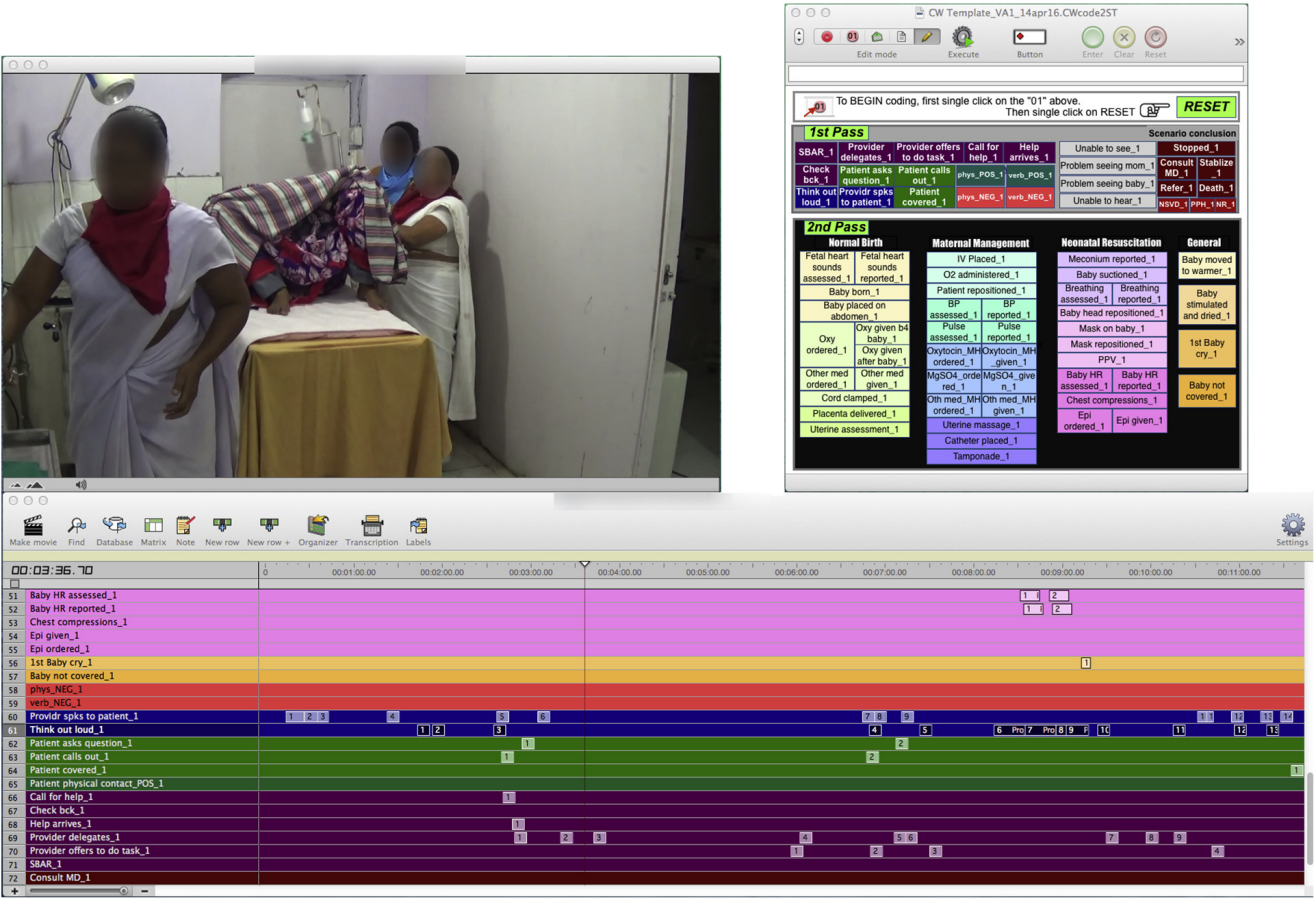
Simulation video analysis code window and timeline.

Building the video analysis system began with selecting key simulation scenarios and agreeing on associated indicators. A team of clinical, simulation, and team training experts agreed on technical and nontechnical indicators. These indicators were incorporated into code windows, the visual coding interface that displays different codes besides the video being analyzed using Studiocode™ video analysis software (Vosaic, Inc., Lincoln, NE) (Figure 3). Expert input and review ensured early identification of coding errors and clarification of indicator definitions, thereby strengthening reliability of coding and overall data quality.

### Approach to Analysis

#### Coding Simulation Videos

Selected videos for analysis fell into two time point categories: (a) midassessment and postassessment videos, which evaluated changes over time in mentees' use of evidence-based practices (EBPs) and their ability to identify and manage simulated maternal and neonatal birth complications in NSVD, PPH, and NR scenarios and (b) simulation videos taken during weeks 3, 5, and 7 of mentoring, allowing program staff to give iterative feedback about trends over time in use of technical and nontechnical skills. For weekly simulations, NMs were encouraged to conduct at least three simulations: NSVD, PPH, and NR, but could exceed this and run any of the 31 simulated scenarios included in the curriculum.

To assess mentee performance during simulations, coding occurred in two passes (Figure 3). During the first pass, VAs coded the nontechnical techniques used. During the second pass, VAs coded clinical EBPs used. While coding for clinical EBPs, a distinction was made between events that were and were not time sensitive:

• Events that occurred repeatedly throughout a simulation were coded for frequency of occurrence (i.e., number of times a provider spoke to a patient).
• Time-bound events were those in which there was an event that should follow a previous event in a specific amount of time (i.e., cord clamping after baby is born). VAs pressed a button to automatically calculate the time between the two events.

Data quality indicators were also recorded on the code window to reflect when the VAs were unable to hear sufficiently or when the camera was blocked and they were unable to see during the video. In addition, a scenario conclusion indicator was included to record the status of the patient at the end of the scenario as stabilized, referred, further consult needed, died, or scenario stopped.

#### Coding Debrief Videos

To assess the quality of debriefs conducted by NMs and provide feedback, a random subset of videos was selected for analysis. Two tools determined the indicators for the debrief videos:

1. Debriefing Assessment for Simulation in Healthcare© (DASH, Center for Medical Simulation, Boston, MA) (Simon, Raemer, Rudolph, 2012): The DASH tool was adapted to rate the quality of debriefing in five key areas by examining the behaviors of the debriefer. A group of clinical, simulation, and research experts with experience working in low-resource settings modified the tool. This questionnaire was completed by VAs after viewing the debrief video for the first time through Qualtrics (www.qualtrics.com/), a web-based survey.
2. Center for Advanced Pediatric and Perinatal Education (CAPE, CAPE Center, Stanford, CA) Debriefing Evaluation Tool© (Center for Advanced Pediatric & Perinatal Education [CAPE], n.d.): VAs used a modified CAPE tool to code for the following actions: (a) arc of components included in the debrief; (b) number of cognitive, technical, and behavioral learning objectives discussed; (c) speaking ratio of number of instructor questions to number of instructor statements; and (d) speaking ratio of number of trainee questions or statements to number of instructor questions or statements. The CAPE tool was adapted into a code window using Studiocode™, which the VAs coded after viewing the debrief video for the second time.

### Interrater Reliability

To ensure consistency of coding and maintain a high level of data quality across the VAs, the research team initially piloted the code windows by triple coding 10 videos. Researchers routinely assessed interrater reliability (IRR) across the two VAs. For each phase, a randomly selected sample of 5% to 10% of the simulation videos was blindly double coded by both VAs and sent to UCSF where IRR scores were calculated in Studiocode™ software. IRR scores were calculated for all indicators individually and averaged for first-pass nontechnical codes and second-pass technical codes. All debrief videos were double coded. The Table describes the IRR for technical and nontechnical indicators by phase.

**Table:** Simulation and Debrief Video Data for Four Phases of a Nurse Mentoring Program in Bihar, India

	Phase 1	Phase 2	Phase 3	Phase 4	Total
Facilities (N)	80	80	80	80	320
Simulation videos					
Per week (N)	1,581	1,555	1,001	869	5,006
Midpoint assessment (N)	N/A	185	240	237	662
Postassessment (N)	102	235	237	295	869
Coded (N)	624	534	434	532	2,124
Double coded (N)	78	39	46	20	183
Technical IRR score (%)	96	93	94	91	94
Nontechnical IRR score (%)	86	81	77	74	80
Debrief videos					
Long debriefs (N)	1,086	1,523	900	778	4,287
Rapid debriefs (N)	163	125	106	60	454
Double coded (N)	55	53	46	62	216

*Note.* IRR = interrater reliability; N/A = not applicable.

### Ethical Issues

The overall study design, including the video recordings of each simulation and debrief in Bihar, was approved by the Committee on Human Research at UCSF under study approval no. 14-15446 and the institutional review board at the Indian Institute of Health Management Research (www.iihmr.org) in Jaipur, India. Written informed consent was obtained from all participating NMs and mentees.

## Results

Throughout the four phases of the nurse midwife mentoring program, a total of 11,278 simulation and debrief videos were collected, resulting in an estimated 2,828 hours of video data as described in the Table. Of this, an estimated 496 hours were analyzed (18% of the total) with 424 hours of video data from simulations and 72 hours from debrief videos. Of the simulation videos, 5,006 were week-wise data exceeding the minimum requirement on an aggregate-level program wide. Some facilities surpassed the minimum requirement, whereas others fell short. A total of 2,124 simulation videos were coded, 183 of which were blindly double coded, equating to 12% of the overall total. Of the coded simulations, all 662 midassessment simulations and all 869 postassessment simulations were coded. For the double-coded sample of 183 simulation videos, the average IRR score was 81% for nontechnical indicators and 94% for technical indicators. Among 4,287 long debrief videos received, 216 were selected and double coded.

Overall, data quality of simulation videos was very good regarding recorded instances of unable to see and unable to hear in phases 1 and 2 of mentoring. Twenty percent of videos recorded one or more instances of being unable to see, whereas inability to see the baby was twice as common as inability to see the mother. In 2% of simulations, VAs reported that they were unable to hear. Video analysis showed that 60% of videos resulted in a stabilized patient at the conclusion of the simulation and 25% were reportedly stopped before the end of the simulation. Reasons for stopping a simulation included needing to attend to a live patient, clinical mismanagement of the simulated case offering the opportunity for a teachable moment, or technical issues such as equipment malfunction.

### Programmatic Decision-Making

The video monitoring system allowed program implementers to use data to guide programmatic decision-making. Because this program rolled out in four phases, data-informed improvements shaped as lessons and were learned on a phase-by-phase basis. For example, after analyzing phase 1 simulation video data, the program implementers recognized NR as an area for knowledge and skill improvement. Feedback was given to enhance the NM's teaching of NR skills by repeating relevant simulations and by providing a timer to sensitize to the importance of urgency. Secondary analysis of these NR videos revealed the difficulty in assessing effective resuscitation, resulting in a set of new instructions about camera placement. A secondary code window was developed for NR experts to better assess the quality of specific NR practices in birth asphyxia simulations.

### Personalized Feedback

**Figure 4:**
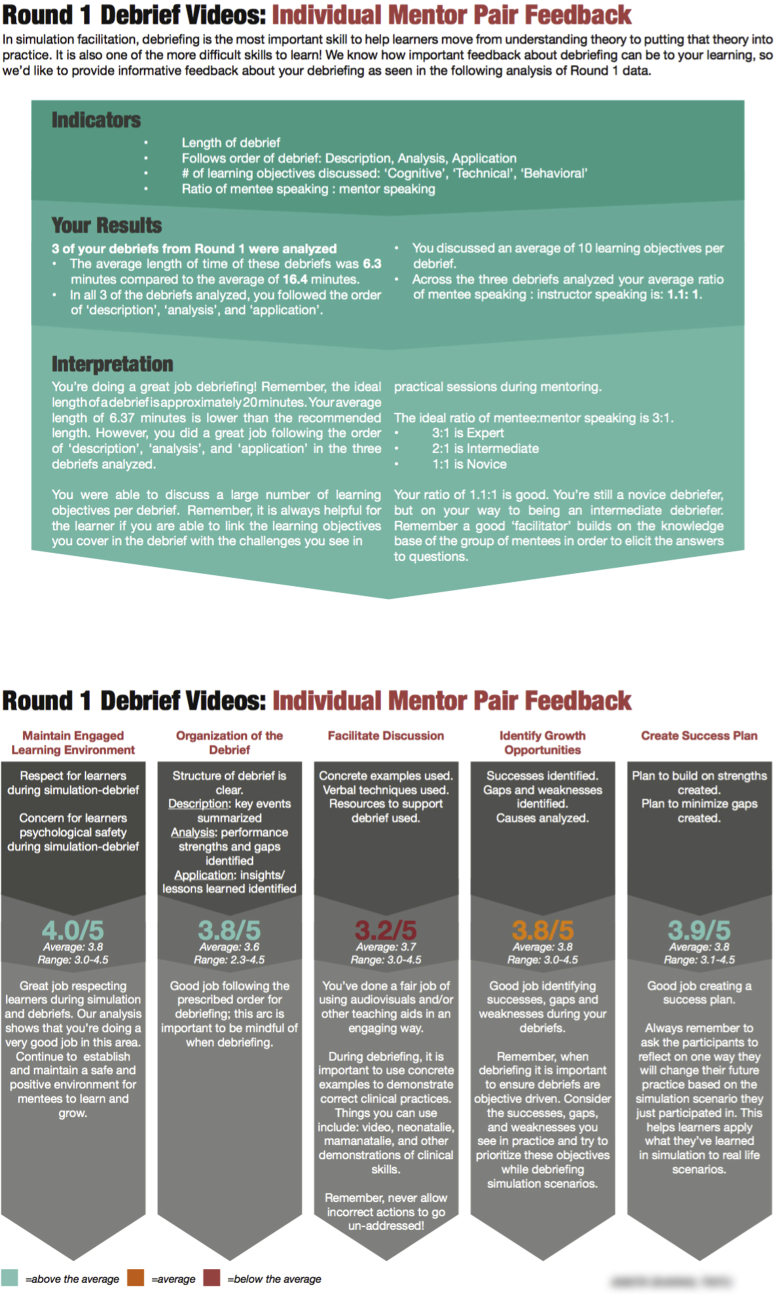
Example of Nurse Mentor (NM) pair debrief feedback.

To ensure mentees received the best training possible, a robust feedback mechanism was developed for NMs about their skills in simulation facilitation and debriefing. The data generated from the video monitoring system were used to develop individualized reports for each NM pair. Reports from simulated cases displayed graphs of mentee performance from previous phases during simulations of NSVD, PPH, and NR. A written interpretation accompanied each graph congratulating NMs on areas in which mentees consistently performed well while encouraging NMs to focus teaching in areas where mentees displayed room for improvement. To give debriefing feedback to NM pairs, program implementers used data collected from the modified CAPE and DASH tools. Figure 4 shows an example of an NM debrief report.

Furthermore, to ensure reports would strengthen individual-specific teaching, program implementers contacted each NM pair either telephonically or in person to discuss the reports and methods in the hope that this would help NMs enhance their facilitation practices.

## Discussion

To our knowledge, this is the first report using video analysis for monitoring and evaluating a maternal neonatal care quality improvement project at this scale and in a resource-limited setting. This article documents the experience of developing and implementing a video monitoring system for clinical quality improvement and demonstrates that video analysis is not only feasible but also a programmatically valuable technique for program monitoring and evaluation at scale in a resource-limited setting. Overall, the video monitoring system implemented was a successful mechanism at providing important feedback to improve and adapt the program on an iterative basis. The system provided data to allow program implementers to measure the use of technical and nontechnical indicators occurring during simulation, supply objective information to guide programmatic decision-making, address technical problems in simulations early, and provide comprehensive feedback to NMs to effectively guide their own simulation facilitation practices.

This approach of analyzing nontechnical behavioral indicators warrants highlighting. Evidence is building for the importance of nontechnical skills for effective clinical care and avoiding errors (Armour Forse et al., 2011, Mayer et al., 2011, Weaver et al., 2014). However, data from team training in low-resource settings are limited (Deering et al., 2011, Merién et al., 2010). This system allowed program implementers to remotely capture and analyze data on nontechnical behavioral practices used by mentees during simulated cases. PRONTO built teamwork and communication into their training strategy as a core component by integrating concepts from Team Strategies and Tools to Enhance Performance and Patient Safety. In Bihar, PRONTO developed and integrated a total of 30 customized activities that promoted teamwork and communication techniques, such as leadership, listening, communication concepts, mutual support, and problem solving. Data generated through video coding and analysis were important to gauge the uptake of these nontechnical skills, allowing program implementers to assess changes in the use of teamwork and communication techniques in simulated cases progressively.

### Challenges and Limitations

Using video recordings for monitoring and evaluation posed challenges. The process of data collection, coding, and transferring large video files was labor intensive. Although programmatic feedback to NMs was iterative, real-time feedback was not possible. Fortunately, NMs served as mentors for two or more phases of implementation, allowing program implementers to use data from past rounds to subsequently develop relevant individualized program reports. In addition, we have not provided data on the cost-benefit or cost-effectiveness of this strategy, which is important for scale-up interventions.

These findings have important implications for scale-up of innovative interventions within government systems. Pilot interventions are often difficult to scale-up (Simmons et al., 2006), hindered by issues such as intervention complexity, limited resources, insufficient leadership, poor management, inadequate health systems capacity, and lack of ownership (Simmons et al., 2006). In addition, existing government monitoring and evaluation systems are seldom able to provide the information needed to assess the scaling-up process while the program is being implemented (Simmons et al., 2006). Our unique approach to monitoring and evaluation bridged this gap, allowing implementers to monitor activities remotely and ensure new and difficult training components were being delivered effectively. It also allowed program implementers to make data-informed adjustments to inform and strengthen future intervention rounds and maintain the fidelity of the intervention, which can be lost when interventions are taken to scale (Simmons et al., 2006).

## Conclusion

This study demonstrates that video monitoring systems can be effectively implemented at scale in resource-limited settings. Furthermore, video monitoring systems can play several vital roles within program implementation, including monitoring and evaluation, provision of actionable feedback to program implementers, and assurance of program fidelity.

## References

[bib1] Armour Forse R., Bramble J.D., McQuillan R. (2011). Team training can improve operating room performance. Surgery.

[bib2] Bragard I., Farhat N., Seghaye M.-C., Karam O., Neuschwander A., Shayan Y., Schumacher K. (2016). Effectiveness of a high-fidelity simulation-based training program in managing cardiac arrhythmias in children. Pediatric Emergency Care.

[bib3] Center for Advanced Pediatric & Perinatal Education (CAPE). (n.d.). Retrieved from http://cape.stanford.edu/programs/for-healthcare-instructors/online-debriefing-assessment.html

[bib4] Das A., Nawal D., Singh M.K., Karthick M., Pahwa P., Shah M.B., Chaudhuri I. (2016). Impact of a nursing skill-improvement intervention on newborn-specific delivery practices: An experience from Bihar, India. Birth.

[bib5] Davis D., O'Brien M.A., Freemantle N., Wolf F.M., Mazmanian P., Taylor-Vaisey A. (1999). Impact of formal continuing medical education: Do conferences, workshops, rounds, and other traditional continuing education activities change physician behavior or health care outcomes?. JAMA.

[bib6] Deering S., Rosen M.A., Ludi V., Munroe M., Pocrnich A., Laky C., Napolitano P.G. (2011). On the front lines of patient safety: Implementation and evaluation of team training in Iraq. Joint Commission Journal on Quality and Patient Safety.

[bib7] District Level Household and Facility Survey (DLHS-4), 2012-2013 (2014). http://rchiips.org/.

[bib8] Kim S., Shin G. (2016). Effects of nursing process-based simulation for maternal child emergency nursing care on knowledge, attitude, and skills in clinical nurses. Nurse Education Today.

[bib9] Levett-Jones T., Lapkin S., Shin S., Schremmer R., Smith K., Harrison M. (2014). A systematic review of the effectiveness of simulation debriefing in health professional education. Nurse Education Today.

[bib10] Mayer C.M., Cluff L., Lin W.-T., Willis T.S., Stafford R.E., Williams C., Amoozegar J.B. (2011). Evaluating efforts to optimize TeamSTEPPS implementation in surgical and pediatric intensive care units. Joint Commission Journal on Quality and Patient Safety.

[bib12] Merién A.E.R., van de Ven J., Mol B.W., Houterman S., Oei S.G. (2010). Multidisciplinary team training in a simulation setting for acute obstetric emergencies: A systematic review. Obstetrics and Gynecology.

[bib13] MMR Bulletin Sample Registration System, 2013 MMR Bulletin Sample Registration System (2013). http://www.censusindia.gov.in/vital_statistics/mmr_bulletin_2011-13.pdf.

[bib21] MMR Bulletin: Primary Census Abstract Data Highlights - 2011 India & States. (Vol. 1). (2011). Delhi, India. Retrieved from http://www.censusindia.gov.in/2011census/PCA/PCA_Highlights/pca_highlights_file/India/5Figures_at_glance.pdf

[bib14] Sawyer T., Eppich W., Brett-Fleegler M., Grant V., Cheng A. (2016). More than one way to debrief: A critical review of healthcare simulation debriefing methods. Simulation in Healthcare: The Journal of the Society for Simulation in Healthcare.

[bib15] Simmons R., Shiffman J., Innovation T., Hoffman L.J., Gill B., America L., Ghiron L. (2006). Beginning with the end in mind. American Journal of Nursing.

[bib16] Simon R., Raemer D.B., Rudolph J.W. (2012). Debriefing Assessment for Simulation in Healthcare (DASH)©—Instructor version long form. https://harvardmedsim.org/_media/DASH.IV.LongForm.2012.05.pdf.

[bib17] Tripathi V., Stanton C., Strobino D., Bartlett L., Porignon D., Velazco A. (2015). Development and validation of an index to measure the quality of facility-based labor and delivery care processes in Sub-Saharan Africa. PLoS One.

[bib18] Walker D.M., Cohen S.R., Fritz J., Olvera-Garcia M., Zelek S.T., Fahey J.O., Lamadrid-Figueroa H. (2016). Impact evaluation of PRONTO Mexico: A simulation-based program in obstetric and neonatal emergencies and team training. Simulation in Healthcare: The Journal of the Society for Simulation in Healthcare.

[bib19] Walker D., Cohen S., Fritz J., Olvera M., Lamadrid-Figueroa H., Cowan J., Fahey J.O. (2014). Team training in obstetric and neonatal emergencies using highly realistic simulation in Mexico: Impact on process indicators. BMC Pregnancy and Childbirth.

[bib20] Weaver S.J., Dy S.M., Rosen M.A. (2014). Team-training in healthcare: A narrative synthesis of the literature. BMJ Quality & Safety.

